# Drooling disrupts the brain functional connectivity network in Parkinson's disease

**DOI:** 10.1111/cns.14251

**Published:** 2023-05-05

**Authors:** Ting Huang, Li‐Li Tang, Jin‐Ying Zhao, Song‖an Shang, Yu‐Chen Chen, You‐Yong Tian, Ying‐Dong Zhang

**Affiliations:** ^1^ Department of Neurology, Nanjing First Hospital Nanjing Medical University Nanjing China; ^2^ Department of Neurology Nanjing Hospital of Chinese Medicine affiliated to Nanjing University of Chinese Medicine Nanjing China; ^3^ Department of Medical Imaging Center, Clinical Medical College Yangzhou University Yangzhou China; ^4^ Department of Radiology, Nanjing First Hospital Nanjing Medical University Nanjing China

**Keywords:** drooling, granger causality analysis, independent component analysis, Parkinson's disease, sensorimotor network

## Abstract

**Aims:**

This study aimed to investigate the causal interaction between significant sensorimotor network (SMN) regions and other brain regions in Parkinson's disease patients with drooling (droolers).

**Methods:**

Twenty‐one droolers, 22 PD patients without drooling (non‐droolers), and 22 matched healthy controls underwent 3T‐MRI resting‐state scans. We performed independent component analysis and Granger causality analysis to determine whether significant SMN regions help predict other brain areas. Pearson's correlation was computed between imaging characteristics and clinical characteristics. ROC curves were plotted to assess the diagnostic performance of effective connectivity (EC).

**Results:**

Compared with non‐droolers and healthy controls, droolers showed abnormal EC of the right caudate nucleus (CAU.R) and right postcentral gyrus to extensive brain regions. In droolers, increased EC from the CAU.R to the right middle temporal gyrus was positively correlated with MDS‐UPDRS, MDS‐UPDRS II, NMSS, and HAMD scores; increased EC from the right inferior parietal lobe to CAU.R was positively correlated with MDS‐UPDRS score. ROC curve analysis showed that these abnormal ECs are of great significance in diagnosing drooling in PD.

**Conclusion:**

This study identified that PD patients with drooling have abnormal EC in the cortico‐limbic‐striatal‐cerebellar and cortio‐cortical networks, which could be potential biomarkers for drooling in PD.

## Graphical abstract

Parkinson's disease patients with drooling (droolers) have changes in the cortico‐limbic‐striatal‐cerebellar and cortico‐cortical networks, which might reflect that droolers have abnormal brain integration functions, neurological, and downregulation or compensation mechanisms in specific areas.

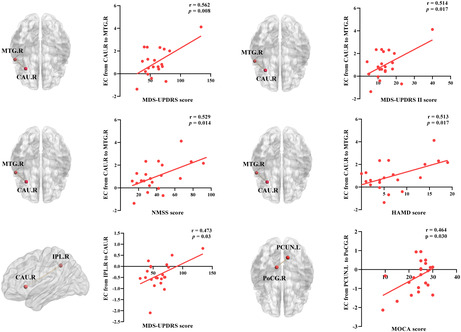

## 1 INTRODUCTION

Drooling, also known as sialorrhea or ptyalism, is defined as excessive saliva that exceeds the edge of the lips. It is a devastating and debilitating complication of Parkinson's disease (PD) that can be caused by excessive saliva production or swallowing dysfunction.^1^ Drooling can occur at any stage of PD, with a prevalence ranging from 9.26 to 70%.^2^ It may cause feelings, such as embarrassment, malodor, and skin infection, and further lead to a lack of self‐confidence, depression, and social isolation for PD patients. In addition to seriously reducing the quality of life of PD patients, these symptoms place a great burden on caregivers. More seriously, the accumulation of secretions in the oropharynx of PD patients considerably increases the risk of aspiration pneumonia, thereby contributing to the morbidity and mortality of aspiration pneumonia in PD patients.^3^ Numerous researchers have recently focused on the prevalence of drooling, associated clinical symptoms, and treatment.^2^, ^4^ However, the cerebral mechanisms of drooling in PD remain unclear.

Recently, resting‐state functional MRI (rsfMRI) has emerged as a novel noninvasive tool that greatly contributes to capturing changes in PD‐related functional connectivity (FC) without requiring subjects to perform specific tasks.^5^ This not only promotes a deeper understanding of PD pathogenesis but also further demonstrates the significant clinical value of neuroimaging biomarkers in PD diagnosis and monitoring. To date, only one rsfMRI study has explored the aberrant FC of the putamen within the bilateral sensorimotor cortices, superior and inferior parietal lobules, and areas in the right occipital and temporal lobes in early‐stage PD patients with drooling (PD‐DR; droolers).^6^ Therefore, we are still far from fully understanding the functional changes in the brain associated with drooling in PD.

Swallowing saliva is a complex process. The somatosensory cortex plays an important role in the execution of swallowing as well as the integration of sensorimotor information related to preparation for swallowing.^7^ The major cause of drooling in PD patients is swallowing dysfunction in the oral and/or pharyngeal phase of swallowing, which is also a manifestation of motor difficulties in PD.^4^ It is reported that motor difficulties in PD are attributed to sensorimotor dysfunction caused by denervation along the cortex‐striatum pathway following nigrostriatal dopaminergic loss.^8^, ^9^, ^10^ Thus, the occurrence of drooling in PD patients is closely associated with sensorimotor areas of the brain. The sensorimotor network (SMN) consists of the primary sensorimotor cortex, secondary somatosensory cortices, and supplementary motor area.^5^ It is crucial for detecting and processing sensory input as well as preparing and executing motor actions. Research has shown that FC within the SMN (premotor cortex, supplementary motor area, and primary motor cortex) is significantly increased in PD patients with dysphagia compared to those without dysphagia.^11^ These findings suggest that altered FC in the SMN may be responsible for the occurrence of drooling in PD.

The present study aimed to investigate alterations in the SMN between droolers and non‐droolers and the causal relationship between significant SMN regions and other brain regions. To extract the SMN, we employed independent component analysis (ICA), a mathematical technique that maximizes statistical independence among its components. In contrast to seed‐based methods, the ICA approach proceeds without a priori seed selection, thus removing the bias from analysis.^12^ Additionally, we performed Granger causality analysis (GCA) using the significant SMN regions as seeds to determine whether the SMN time series could be useful for predicting other brain areas. The GCA's capacity to predict the direction of information flow in brain networks has led to its widespread application.^13^ We hypothesized that: (1) droolers would show abnormal intrinsic connectivity within the SMN relative to PD patients without drooling (PD‐NDR; non‐droolers) and (2) effective connectivity (EC) between significant SMN regions as seeds and other brain regions would be further detected, which correlates with clinical scores.

## 2 MATERIALS AND METHODS

### 2.1 Subjects

A total of 43 consecutive individuals with idiopathic PD and 22 age‐ and gender‐matched healthy controls (HCs) were included in this study (Table 1). These PD patients were recruited via the movement disorders outpatient clinic of Nanjing First Hospital and diagnosed by experienced neurologists according to the 2015 Movement Disorder Society clinical diagnostic criteria for PD.^14^ Individuals without drooling enrolled in the HCs group were cognitively normal, free of neurological and psychiatric disorders, and had no family history of PD. For the exclusion criteria for all the participants, see Supplemental Materials. The human participant studies were reviewed and approved following the Declaration of Helsinki and the institutional review board of Nanjing First Hospital. All study participants provided their written informed consent for this study.

**TABLE 1:** Demographic and clinical characteristics of the total sample.

Characteristics	PD‐DR (*n* = 21)	PD‐NDR (*n* = 22)	HCs (*n* = 22)	*p* value^a^	*p* value^b^
Age (years)	71.93 ± 8.30	68.60 ± 7.13	68.67±7.66	0.278	–
Gender (M/F)	15/6	11/11	9/13	0.121	–
Handedness of writing (R: L)	21:0	22:0	22:0	–	–
Education (years)	8.62 ± 4.94	11.00 ± 4.84	9.64 ± 4.29	0.256	–
Disease duration (years)	6.57 ± 5.74	4.36 ± 2.29	–	–	0.113
LEDD (mg/day)	377.05 ± 232.32	311.52 ± 198.97	–	–	0.326
H‐Y stage	2.43 ± 0.68	2.11 ± 0.65	–	–	0.128
MDS‐UPDRS score	60.05 ± 22.21	40.77 ± 20.98	–	–	0.006*
MDS‐UPDRS I score	7.86 ± 5.33	5.45 ± 4.78	–	–	0.127
MDS‐UPDRS II score	13.86 ± 7.59	6.86 ± 4.72	–	–	0.001*
MDS‐UPDRS III score	37.81 ± 12.90	27.91 ± 14.93	–	–	0.025*
MDS‐UPDRS IV score	0.52 ± 1.834	0.55 ± 1.60	–	–	0.967
NMSS score	39.38 ± 21.58	22.95 ± 18.71	–	–	0.011*
NMSS‐19 domain score	3.81 ± 1.08	0.09 ± 0.43	–	–	0.000*
SCS‐PD score	7.90 ± 3.55	0.00	–	–	0.000*
MMSE score	26.86 ± 2.95	27.73 ± 2.80	27.95 ± 2.36	0.386	0.327
MOCA score	21.38 ± 4.85	23.59 ± 5.73	23.86 ± 2.66	0.162	0.181
HAMD score	7.00 ± 5.62	6.91 ± 6.00	–	–	0.959
HAMA score	6.81 ± 4.84	5.73 ± 5.52	–	–	0.499

Abbreviations: HAMA score, Hamilton Anxiety Scale score; HAMD score, Hamilton Depression Scale score; HCs, healthy controls; H‐Y stage, modified Hoehn‐Yahr stage; LEDD, levodopa equivalent daily dose; MDS‐UPDRS I score, Movement Disorder Society‐Unified Parkinson's Disease Rating Scale Part I score; MDS‐UPDRS II score, Movement Disorder Society‐Unified Parkinson's Disease Rating Scale Part II score; MDS‐UPDRS III score, Movement Disorder Society‐Unified Parkinson's Disease Rating Scale Part III score; MDS‐UPDRS IV score, Movement Disorder Society‐Unified Parkinson's Disease Rating Scale Part IV score; MDS‐UPDRS score, Movement Disorder Society‐Unified Parkinson's Disease Rating Scale total score; MMSE score, Mini‐Mental State Examination score; MOCA score, Montreal Cognitive Assessment score; NMSS score, Non‐Motor Symptom Scale score; PD, Parkinson's disease; PD‐DR, PD patients with drooling; PD‐NDR, PD patients without drooling; SCS‐PD score, Sialorrhea Clinical Scale for Parkinson's disease score.

* Indicates significant difference.

^a^ Comparison among PD‐DR, PD‐NDR, and HCs.

^b^ Comparison between PD‐DR and PD‐NDR.

### 2.2 Study design

Item number 2 of the Movement Disorder Society‐Unified Parkinson's Disease Rating Scale (MDS‐UPDRS) part II was used to evaluate drooling in PD. Patients with a score ≥3 were classified as “droolers,” and those with a score <1 were classified as “non‐droolers.” Thus, all enrolled participants were divided into three groups, PD‐DR (*n* = 21), PD‐NDR (*n* = 22), and HCs (*n* = 22), and underwent clinical data collection in the “off” medication state after MRI scanning (Supplemental Materials). Two experienced neurologists blinded to the MRI data performed clinical examinations and recorded clinical data.

### 2.3 fMRI data acquisition

MRI scans were performed using a 3.0‐Tesla MR imaging system (MAGNETOM Prisma; Siemens Healthcare, Erlangen, Germany) with a 64‐channel receiver array head coil. Functional imaging data based on BOLD were acquired using a gradient echo‐planar imaging (EPI) sequence. The sequence parameters are depicted in the Supplemental Materials.

### 2.4 Data preprocessing

The rsfMRI data were preprocessed using the Graph Theoretical Network Analysis Toolbox version 2.0 (GRETNA v2.0, http://www.nitrc.org/projects/gretna/p/). The specific procedure is described in the Supplemental Materials.

### 2.5 Independent component analysis

To identify the SMN in all PD patients, group spatial ICA was performed using the Group ICA 4.0b of the fMRI Toolbox (GIFT, http://mialab.mrn.org/software/gift/; RRID: SCR_001953) based on the MATLAB R2013b platform. Finally, two seed regions of interest (ROIs) were selected for the GCA using the results of ICA two‐sample t‐tests of SMN. The specific steps of ICA are detailed in the Supplemental Materials.

### 2.6 Granger causality analysis

The right caudate nucleus (CAU.R) and right postcentral gyrus (PoCG.R) were used as ROIs for GCA analysis. We further performed time‐series‐relevant GCA using REST software^15^ to investigate the causal interaction between two ROIs and each voxel in the entire brain. We provide a brief description of GCA analysis in the Supplemental Materials.

### 2.7 Statistical analysis

The demographic and clinical data were analyzed using SPSS version 26.0 software (IBM Corp), with the significance threshold set at *p* < 0.05. Categorical variables are presented as counts and percentages, and continuous variables are summarized as the mean and standard deviation [M (±SD)]. A two‐tailed t‐test or one‐way analysis of variance was conducted on all continuous variables (*p* < 0.05).

We used REST to analyze the EC differences between two ROIs (CAU.R and PoCG.R) and other brain regions. Two‐tailed t‐tests were performed to identify between‐group differences in the significant EC, with age, gender, and education years as covariates. A cluster threshold of *p* < 0.01 with AlphaSim correction (single voxel *p* < 0.01, cluster size >40 voxels) was set.

The relationships between EC and clinical variables such as MDS‐UPDRS, Non‐Motor Symptom Scale (NMSS), Sialorrhea Clinical Scale for Parkinson's disease (SCS‐PD), Mini‐Mental State Examination (MMSE), Montreal Cognitive Assessment (MOCA), Hamilton Depression Scale (HAMD), and Hamilton Anxiety Scale (HAMA) scores in droolers and non‐droolers were calculated by Pearson correlation analysis through SPSS software. Two‐sided *p* values <0.05 were considered significant, corrected for age, sex, and education. The Bonferroni correction was applied in the correlation analysis involving multiple comparisons. Additionally, receiver operating characteristic (ROC) curves were plotted to assess the diagnostic performance of EC in droolers, and Youden's J parameter was measured to find the optimum threshold.

## 3 RESULTS

### 3.1 Demographic and clinical characteristics

Sixty‐five subjects were included in this study, including 21 droolers, 22 non‐droolers, and 22 healthy controls. All groups were matched for age, gender, handedness, education, and MMSE and MOCA scores (*p* > 0.05). No significant differences were detected in disease duration; levodopa equivalent daily dose (LEDD); modified Hoehn‐Yahr stage (H‐Y stage); and MDS‐UPDRSI/IV, HAMD, and HAMA scores (between the PD‐DR and PD‐NDR groups (*p* > 0.05). However, the MDS‐UPDRS (*p* = 0.006), MDS‐UPDRS II (*p* = 0.001), MDS‐UPDRS III (*p* = 0.025), NMSS (*p* = 0.011), NMSS‐19 domain (*p* = 0.000), and SCS‐PD (*p* = 0.000) scores in the PD‐DR group were significantly higher than those in the PD‐NDR group. Demographic data and clinical manifestations of participants are summarized in Table 1.

### 3.2 ICA results

**FIGURE 1:**
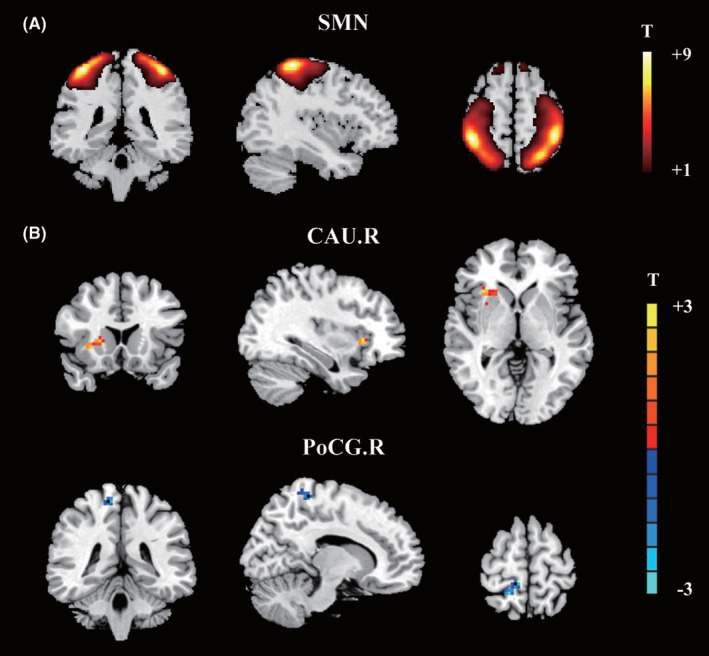
Independent components analysis between PD patients with drooling and PD patients without drooling. (A) Spatial maps of identified SMN; (B) Inter‐group comparison within the SMN revealed the CAU.R with increased activity and PoCG.R with decreased activity. The threshold of cluster *p* was set at <0.05 with AlphaSim correction. CAU.R, right caudate nucleus; PoCG.R, right postcentral gyrus; PD, Parkinson's disease; PD‐DR, PD patients with drooling; PD‐NDR, PD patients without drooling; SMN, sensorimotor network.

According to the ICA analysis, we observed the SMN in the PD‐DR and PD‐NDR groups (Figure 1A). Compared to non‐droolers, droolers showed significantly increased FC values in the CAU.R and significantly reduced FC values in the PoCG.R (Figure 1B). These two brain regions within the SMN extracted from the ICA were selected as ROIs for further ROIwise GCA investigation. The corresponding ROIs are listed in Table 2.

**TABLE 2:** Two ROIs within SMN extracted from the ICA in PD patients with drooling compared with those without drooling.

Brain region (AAL)	BA	MNI coordinates (mm)			Peak T value	Cluster size (mm^3^)
		X	Y	Z		
Right caudate nucleus	13	33	21	0	2.9678	42
Right postcentral gyrus	1	12	−39	66	−3.0092	40

Abbreviations: BA, Brodmann area; ICA, independent component analysis; MNI, Montreal Neurological Institute coordinates; PD, Parkinson's disease; ROIs, seed regions of interest; SMN, sensorimotor network.

### 3.3 ROIwise GCA results

#### 3.3.1 EC from the CAU.R

**FIGURE 2:**
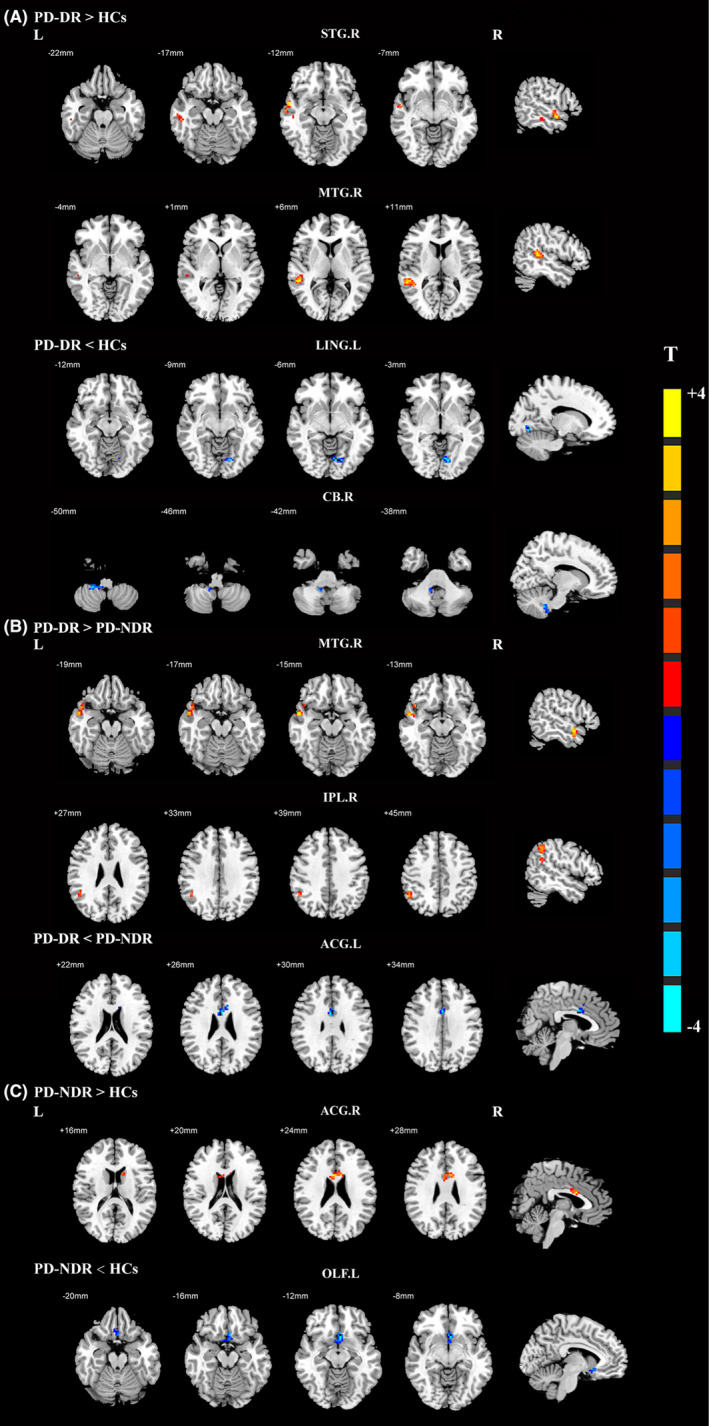
Effective connectivity from the right caudate among PD patients with drooling, PD patients without drooling, and healthy controls. (A) Significant regions between PD patients with drooling and healthy controls; (B) Significant regions between PD patients with drooling and PD patients without drooling; (C) Significant regions between PD patients without drooling and healthy controls. The threshold of cluster *p* was set at <0.01 with AlphaSim correction. The warm color represents the significantly increased effective functional connectivity, and the cold color indicates the significantly decreased effective functional connectivity. ACG.L, left anterior cingulate and paracingulate gyri; ACG.R, right anterior cingulate and paracingulate gyri; CB.R, right cerebellum; HCs, healthy controls; IPL.R, right inferior parietal lobe; LING.L, left lingual gyrus; MTG.R, right middle temporal gyrus; MTG.R, right middle temporal gyrus; OLF.L, left olfactory cortex; PD, Parkinson's disease; PD‐DR, PD patients with drooling; PD‐NDR, PD patients without drooling; STG.R, right superior temporal gyrus.

Compared with healthy controls, droolers showed increased EC from the CAU.R to the right superior temporal gyrus (STG.R) and right middle temporal gyrus (MTG.R) and decreased EC from the CAU.R to the left lingual gyrus (LING.L) and right cerebellum (CB.R) (Figure 2A, Table 3). However, significantly increased EC from the CAU.R to the MTG.R and right inferior parietal lobe (IPL.R) and significantly decreased EC from the CAU.R to the left anterior cingulate and paracingulate gyri (ACG.L) were found in droolers compared with non‐droolers (Figure 2B, Table 3). Additionally, non‐droolers showed increased EC from the CAU.R to the right anterior cingulate and paracingulate gyri (ACG.R) and decreased EC from the CAU.R to the left olfactory cortex (OLF.L) relative to healthy controls (Figure 2C, Table 3).

**TABLE 3:** Effective connections from the right caudate nucleus among the PD‐DR, PD‐NDR, and HCs groups.

Brain region (AAL)	BA	MNI coordinates (mm)			Peak T value	Cluster size (mm^3^)
		X	Y	Z		
PD‐DR > HCs						
Right superior temporal gyrus	22	57	0	−12	4.0942	57
Right middle temporal gyrus	22	51	−39	6	3.9467	110
PD‐DR < HCs						
Left lingual gyrus	18	−15	−75	−6	−4.5834	40
Right cerebellum		12	−48	−42	−3.7249	43
PD‐DR > PD‐NDR						
Right middle temporal gyrus	22	57	0	−15	3.9012	66
Right inferior parietal lobe	39	51	−51	39	3.4321	66
PD‐DR < PD‐NDR						
Left anterior cingulate and paracingulate gyri	24	0	3	30	−4.2784	54
PD‐NDR > HCs						
Right anterior cingulate and paracingulate gyri	24	3	9	24	3.9614	49
PD‐NDR < HCs						
Left olfactory cortex	25	−3	18	−12	−4.3947	67

Abbreviations: BA, Brodmann area; HCs, healthy controls; MNI, Montreal Neurological Institute coordinates; PD, Parkinson's disease; PD‐DR, PD patients with drooling; PD‐NDR, PD patients without drooling.

#### 3.3.2 EC to the CAU.R

**FIGURE 3:**
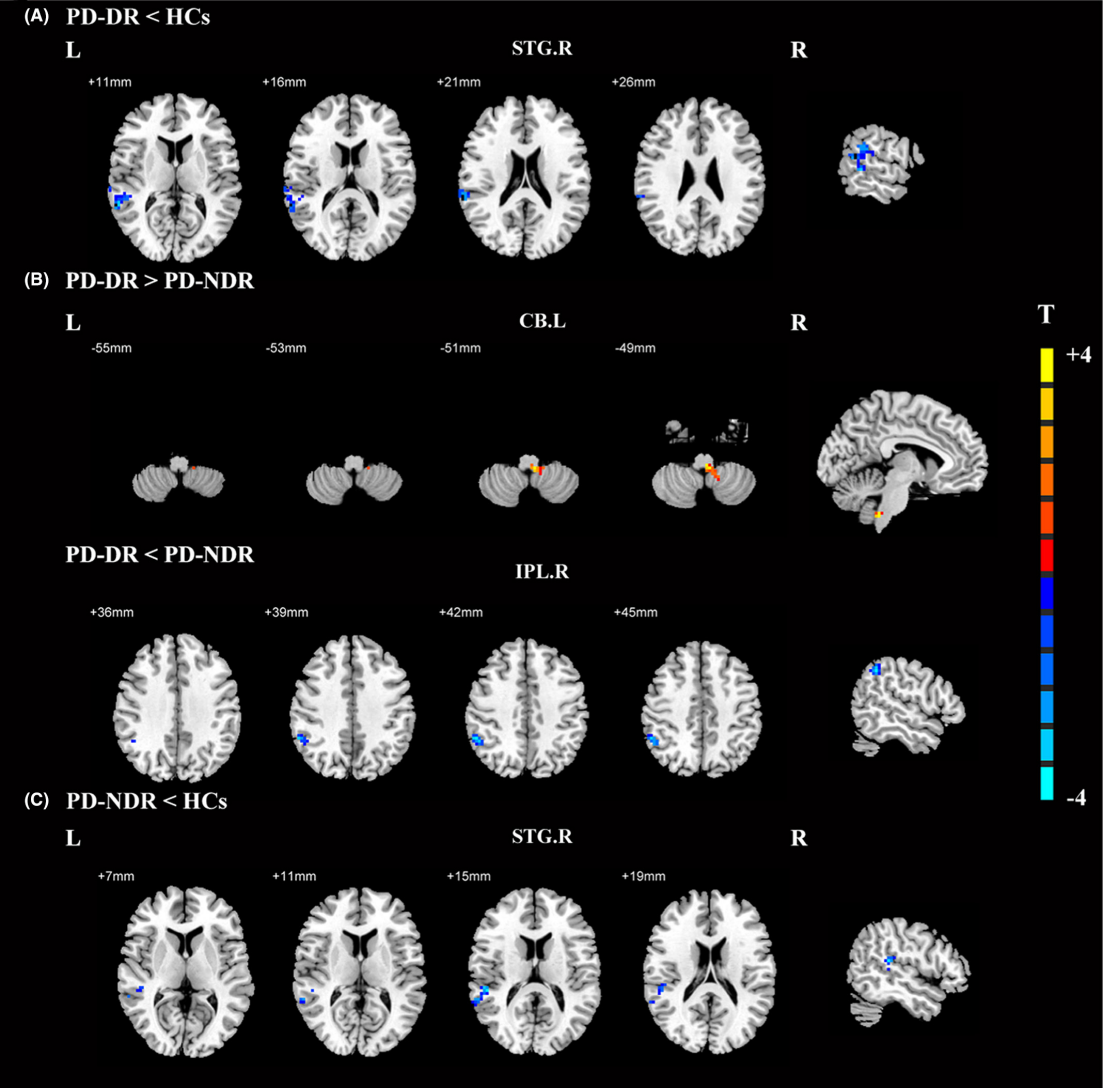
Effective connectivity to the right caudate among PD patients with drooling, PD patients without drooling, and healthy controls. (A) Significant regions between PD patients with drooling and healthy controls; (B) Significant regions between PD patients with drooling and PD patients without drooling; (C) Significant regions between PD patients without drooling and healthy controls. The threshold of cluster *p* was set at <0.01 with AlphaSim correction. The warm color represents the significantly increased effective functional connectivity, and the cold color indicates the significantly decreased effective functional connectivity. HCs, healthy controls; CB.L, left cerebellum; IPL.R, right inferior parietal lobe; PD, Parkinson's disease; PD‐DR, PD patients with drooling; PD‐NDR, PD patients without drooling; STG.R, right superior temporal gyrus.

Compared to healthy controls, we found significantly weakened EC from the STG.R to the CAU.R in both non‐droolers and droolers (Figure 3a,c, Table 4). Compared with non‐droolers, droolers showed significantly strengthened EC from the left cerebellum (CB.L) to the CAU.R; but weakened EC from the IPL.R to the CAU.R (Figure 3b, Table 4).

**TABLE 4:** Effective connections to the right caudate nucleus among the PD‐DR, PD‐NDR, and HCs groups.

Brain region (AAL)	BA	MNI coordinates (mm)			Peak T value	Cluster size (mm^3^)
		X	Y	Z		
PD‐DR < HCs						
Right superior temporal gyrus	22	66	−36	21	−4.2534	127
PD‐DR > PD‐NDR						
Left cerebellum		−6	−42	−51	4.9219	46
PD‐DR < PD‐NDR						
Right inferior parietal lobe	39	54	−48	42	−3.8587	40
PD‐NDR < HCs						
Right superior temporal gyrus	22	51	−36	15	−3.8462	41

Abbreviations: BA, Brodmann area; HCs, healthy controls; MNI, Montreal Neurological Institute coordinate; PD, Parkinson's disease; PD‐DR, PD patients with drooling; PD‐NDR, PD patients without drooling.

#### 3.3.3 EC from the PoCG.R

**FIGURE 4:**
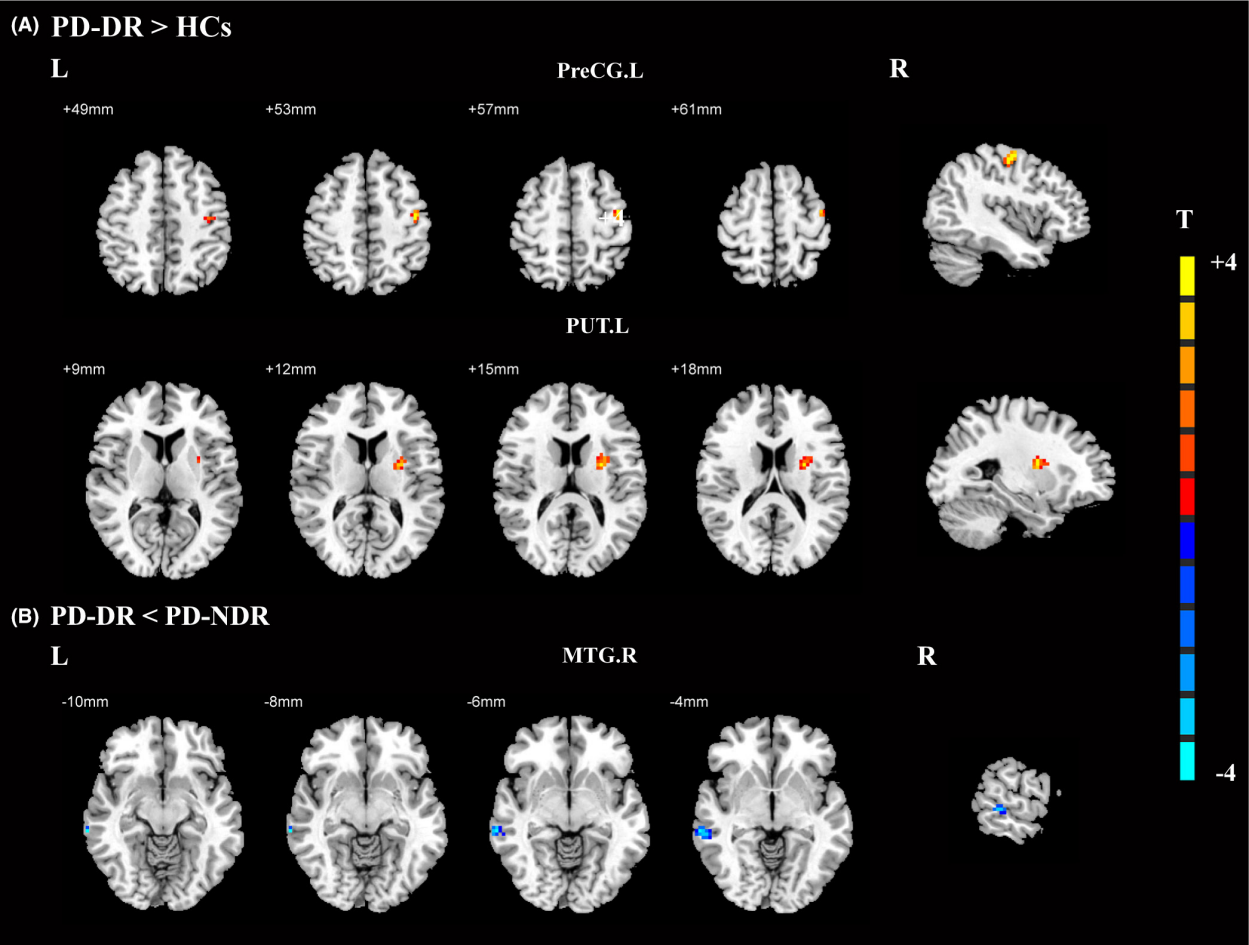
Effective connectivity from the right postcentral gyrus among PD patients with drooling, PD patients without drooling, and healthy controls. (A) Significant regions between PD patients with drooling and healthy controls; (B) Significant regions between PD patients with drooling and PD patients without drooling. The threshold of cluster *p* was set at <0.01 with AlphaSim correction. The warm color represents the significantly increased effective functional connectivity, and the cold color indicates the significantly decreased effective functional connectivity. HCs, healthy controls; MTG.R, right middle temporal gyrus; PreCG.L, left precentral gyrus; PUT.L, Left putamen; PD, Parkinson's disease; PD‐DR, PD patients with drooling; PD‐NDR, PD patients without drooling.

For the causal connectivity from the PoCG.R to other brain regions, droolers exhibited significantly increased causal connectivity from the PoCG.R to the left precentral gyrus (PreCG.L) and left putamen (PUT.L) relative to healthy controls (Figure 4A, Table 5). Additionally, droolers showed significantly decreased causal connectivity from the PoCG.R to the MTG.R compared to non‐droolers (Figure 4B, Table 5). However, no significant differences were found in the causal connectivity from the PoCG.R to other brain regions between non‐droolers and healthy controls.

**TABLE 5:** Effective connections from the right postcentral gyrus among the PD‐DR, PD‐NDR, and HCs groups.

Brain region (AAL)	BA	MNI coordinates (mm)			Peak T value	Cluster size (mm^3^)
		X	Y	Z		
PD‐DR > HCs						
Left precentral gyrus	6	−42	−12	57	4.8807	56
Left putamen	49	−27	−3	15	3.9178	40
PD‐DR < PD‐NDR						
Right middle temporal gyrus	21	66	−33	−6	−3.918	40

Abbreviations: BA, Brodmann area; HCs, healthy controls; MNI, Montreal Neurological Institute coordinates; PD, Parkinson's disease; PD‐DR, PD patients with drooling; PD‐NDR, PD patients without drooling.

#### 3.3.4 EC to the PoCG.R

**FIGURE 5:**
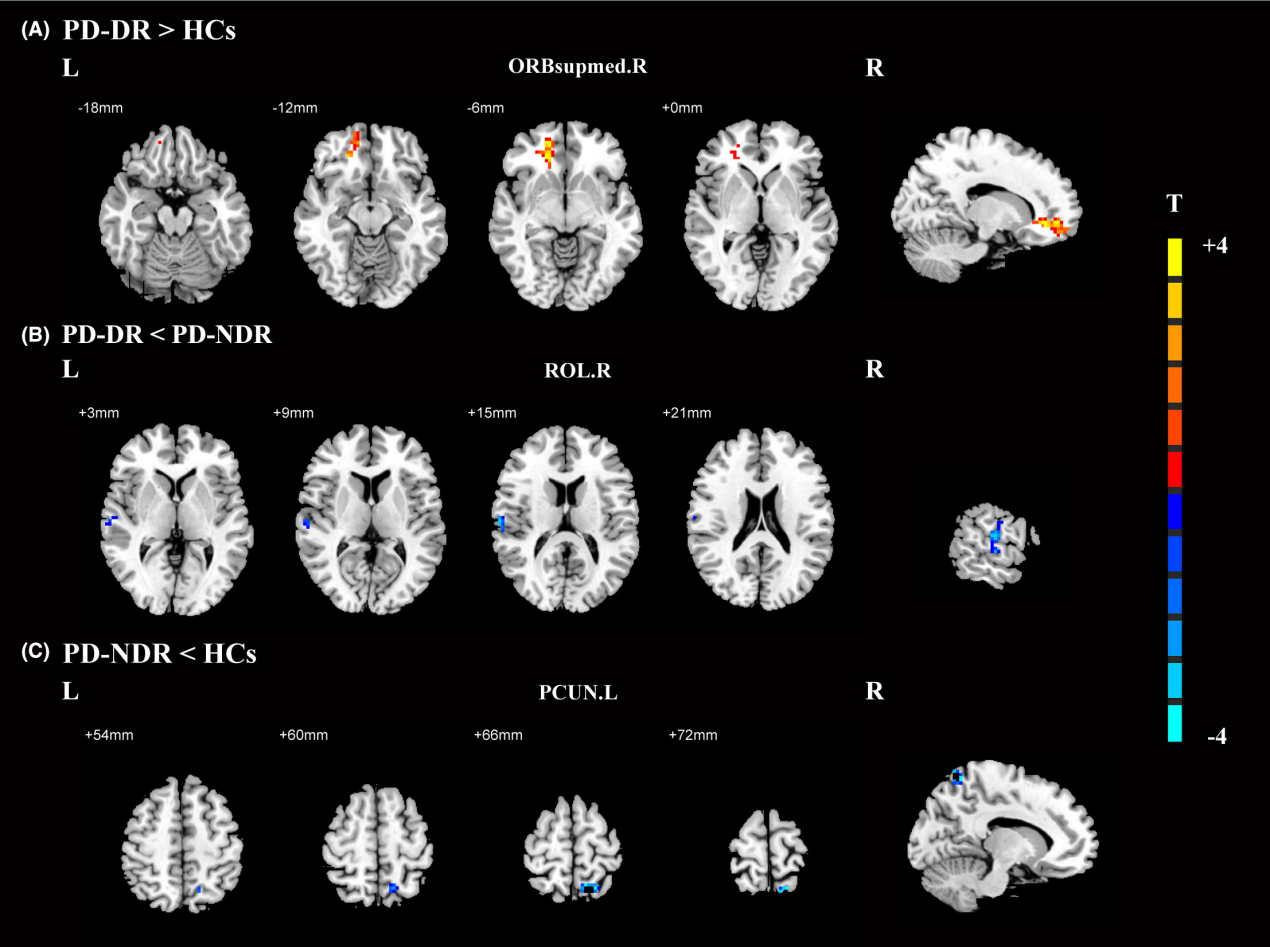
Effective connectivity to the right postcentral gyrus among PD patients with drooling, PD patients without drooling, and healthy controls. (A) Significant regions between PD patients with drooling and healthy controls; (B) Significant regions between PD patients with drooling and PD patients without drooling; (C) Significant regions between PD patients without drooling and healthy controls. The threshold of cluster *p* was set at <0.01 with AlphaSim correction. The warm color represents the significantly increased effective functional connectivity, and the cold color indicates the significantly decreased effective functional connectivity. HCs, healthy controls; ORBsupmed.R, right medial orbital part of superior frontal gyrus; PCUN.L, left precuneus gyrus; PD, Parkinson's disease; PD‐DR, PD patients with drooling; PD‐NDR, PD patients without drooling; ROL.R, right Rolandic operculum.

Compared to healthy controls, droolers showed significantly increased inflow from the right medial orbital part of the superior frontal gyrus (ORBsupmed.R) to the PoCG.R, while non‐droolers showed significantly decreased inflow from the left precuneus gyrus (PCUN.L) to the PoCG.R (Figure 5A,C, Table 6). Compared with non‐droolers, droolers showed significantly decreased inflow from the right Rolandic operculum (ROL.R) to the PoCG.R (Figure 5B, Table 6).

**TABLE 6:** Effective connections to the right postcentral gyrus among the PD‐DR, PD‐NDR, and HCs groups.

Brain region (AAL)	BA	MNI coordinates (mm)			Peak T value	Cluster size (mm^3^)
		X	Y	Z		
PD‐DR > HCs						
Right medial orbital part of superior frontal gyrus	10	15	51	−6	5.0629	97
PD‐DR < PD‐NDR						
Right Rolandic operculum	40	63	−18	15	−3.7281	42
PD‐NDR < HCs						
Left precuneus gyrus	7	−12	−60	66	−5.1226	60

Abbreviations: BA, Brodmann area; HCs, healthy controls; MNI, Montreal Neurological Institute coordinates; PD, Parkinson's disease; PD‐DR, PD patients with drooling; PD‐NDR, PD patients without drooling.

### 3.4 Correlations between abnormal connectivity and clinical characteristics

**FIGURE 6:**
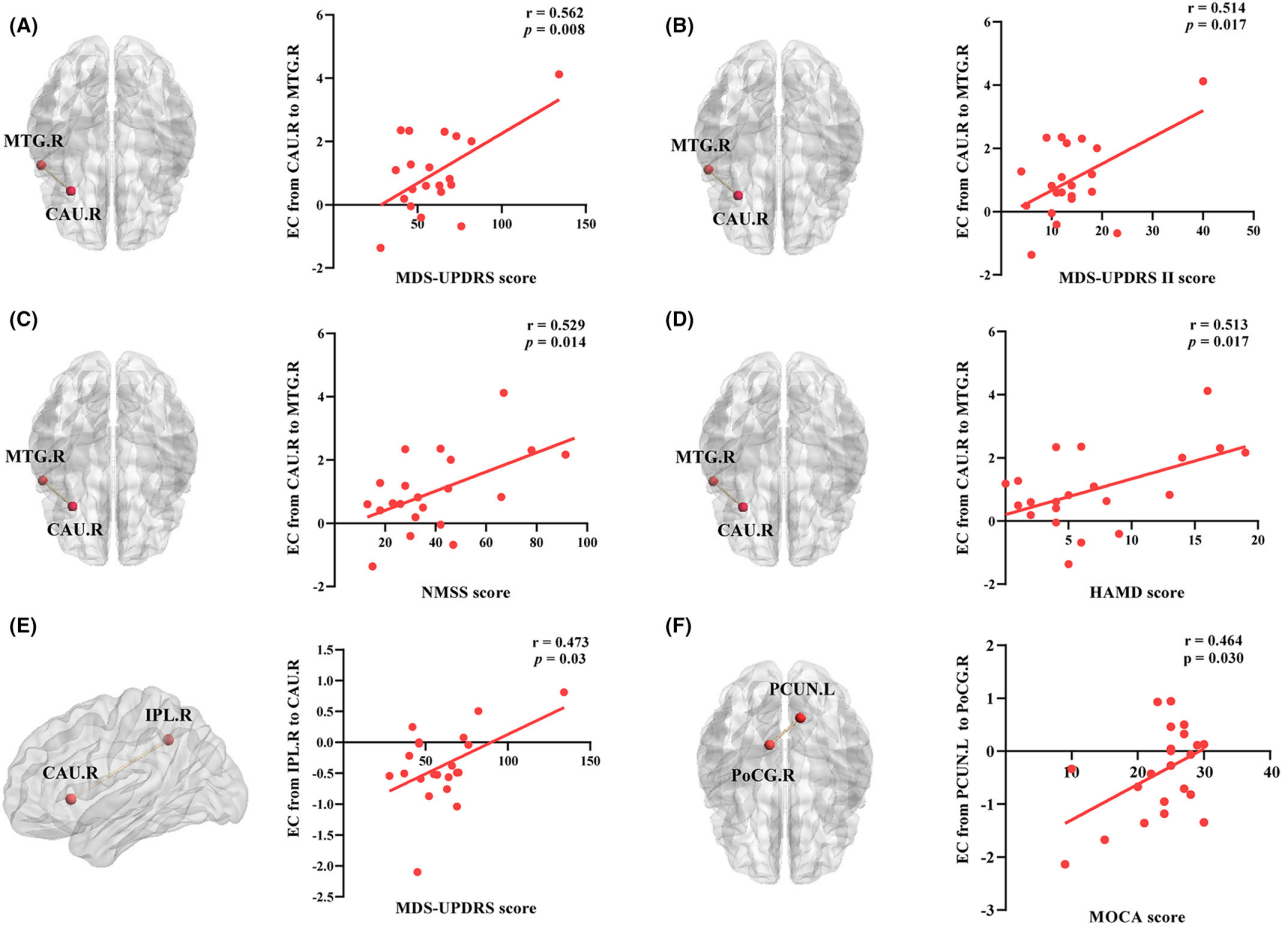
Significant correlations between abnormal connectivity and clinical characteristics in PD patients with drooling and PD patients without drooling. (A–D) The increased connections from the CAU.R to the MTG.R showed a positive correlation with MDS‐UPDRS, MDS‐UPDRS II, NMSS, and HAMD scores in PD patients with drooling; (E) The increased connections from the IPL.R to the CAU.R showed a positive correlation with MDS‐UPDRS score in PD patients with drooling; (F) The increased connections from the PCUN.L to the PoCG.R was positively associated with MOCA score in PD patients without drooling. CAU.R, right caudate nucleus; HAMD score, Hamilton Depression Scale score; IPL.R, right inferior parietal lobe; MTG.R, right middle temporal gyrus; MDS‐UPDRS total score, Movement Disorder Society‐Unified Parkinson's Disease Rating Scale total scores; MDS‐UPDRS II score, Movement Disorder Society‐Unified Parkinson's Disease Rating Scale Part II score; MOCA score, Montreal Cognitive Assessment score; NMSS score, Non‐Motor Symptom Scale; PoCG.R, right postcentral gyrus; PCUN.L, left precuneus gyrus; PD, Parkinson's disease; PD‐DR, PD patients with drooling; PD‐NDR, PD patients without drooling.

Pearson correlation coefficients were used to examine the associations between abnormal connectivity and clinical characteristics in droolers and non‐droolers. For the EC from the CAU.R to other brain regions in the PD‐DR group, the increased connections from the CAU.R to the MTG.R showed a positive correlation with MDS‐UPDRS (*r* = 0.562, *p* = 0.008; Figure 6A), MDS‐UPDRS II (r = 0.514, *p* = 0.017; Figure 6B), NMSS (*r* = 0.529, *p* = 0.014; Figure 6C), and HAMD (*r* = 0.513, *p* = 0.017; Figure 6D) scores. For the EC from other brain regions to the CAU.R in the PD‐DR group, a positive correlation was also found between increased EC from the IPL.R to the CAU.R and MDS‐UPDRS score (*r* = 0.473, *p* = 0.03; Figure 6E). Furthermore, the enhanced EC from the PCUN.L to the PoCG.R was positively associated with the MOCA score in the PD‐NDR group (*r* = 0.464, *p* = 0.030; Figure 6F). After correction for age, sex, and education, significant correlations remained (Table S1). There were no significant associations between abnormal connectivity and these clinical characteristics after the Bonferroni correction. Disrupted EC was not correlated with the NMSS‐19 domain, SCS‐PD, MMSE, or HAMA scores.

### 3.5 ROC analysis for differential diagnosis

ROC analysis was used to evaluate the diagnostic value of EC strength for distinguishing droolers from non‐droolers. For the EC from the CAU.R to the MTG.R, IPL.R, and ACG.L, ROC curve analysis showed that the diagnostic power of the EC values was 0.864, 0.805, and 0.905, respectively (Figure S1). For the EC from the CB.L and IPL.R to the CAU.R, the diagnostic power of the EC values was 0.909 and 0.816, respectively (Figure S1). Additionally, the diagnostic power of the EC values from the PoCG.R to the MTG.R and from the ROL.R to the PoCG.R were 0.836 and 0.823, respectively (Figure S1). The area under the ROC curve (AUC), 95% confidence interval (CI), *p* value, optimal cutoff value, sensitivity, specificity, and Youden index of EC values are shown in Table S2. The results indicate that these connections are useful as biomarkers to distinguish droolers from non‐droolers.

## 4 DISCUSSION

By comparing a cohort of PD patients with and without drooling and healthy controls using rsfMRI, we observed abnormal connectivity in the cortico‐striatal, cerebellar‐striatal, limbic‐striatal, and cortio‐cortical loops in droolers. Increased inflow was positively correlated with the severity of motor symptoms, nonmotor symptoms, and depression in droolers. These abnormal directional connectivities may be of great significance in the differential diagnosis of drooling in PD. We speculated that drooling disrupted the functional integration of the cortico‐limbic‐striatal‐cerebellar and cortio‐cortical networks, resulting in motor function and neuropsychological deficits in PD patients with drooling. These findings provide novel insight into the large‐scale functional reorganization of drooling in PD.

Drooling is a devastating and debilitating complication of PD and other neurological diseases including cerebral palsy and amyotrophic lateral sclerosis. Due to dopamine deficiency, an accumulating body of evidence has indicated that PD patients produce less saliva than healthy controls.^16^, ^17^ Consequently, decreased salivary clearance due to oropharyngeal bradykinesia may be the major cause of drooling in PD patients.^18^, ^19^ A recent meta‐analysis suggested that the higher prevalence of drooling was related to various factors, such as disease severity, bradykinesia, dysphagia, dysarthria, and cognition.^2^ However, fewer studies on the functional brain network of drooling in PD have been reported. Hou et al.^6^ explored the reduced FC of the putamen with the bilateral sensorimotor areas, and the parietal, occipital, and temporal lobes in PD patients with drooling relative to those without drooling. In this study, we also found abnormal connections of the CAU.R and PoCG.R to similar extensive brain regions, including frontal regions (PreCG.L and ORBsupmed.R), temporal regions (STG.R and MTG.R), parietal regions (IPL.R and ROL.R), an occipital region (LING.L), the limbic system (ACG.L), putamen and cerebellum, which may reflect major changes related to drooling in PD and support the view that these regions constitute a “drooling network” in PD.

### 4.1 EC from and to CAU.R

Convergent evidence from prior observations has implicated the striatum in the pathophysiology of drooling in PD.^6^, ^20^ A ^123^I‐FP‐CIT SPECT imaging study revealed that drooling in PD was associated with reduced striatal DAT availability.^20^ In priori ROI analyses, PD patients complaining of drooling exhibited functional disconnection between the putamen and cortical, with remarkable alterations, which indicated that interrupt FC in the cortico‐striatal loop may be a cardinal contributor to the “drooling network” in PD.^6^ As an essential component of the dorsal striatum structure, the caudate nucleus together with the putamen form the primary input nucleus of the basal ganglia in the cortico‐striatal dopaminergic circuitry. However, Hou et al. did not identify abnormal FC between the caudate nucleus and cortical in droolers. Different from the previous research method, we performed the ICA method and found abnormal EC between CAU.R and extensive brain cortex, including temporal regions (STG.R and MTG.R), parietal regions (IPL.R), occipital region (LING.L), the limbic system (ACG.L), and cerebellum. The drooling‐related cortico‐limbic‐striatal‐cerebellar network was influenced distinctly through the caudate nucleus, suggesting that drooling in PD might be explained better by the caudate nucleus pathology.

MTG.R and STG.R belong to the temporal cortex and IPL.R to the prefrontal cortex, and they all belong to the default mode network (DMN) regions. The main role of DMN is to dynamically integrate external inputs with internal prior knowledge, which aids in episodic memory, emotional processing, and social cognition.^21^ In PD, DMN plays a role in both motor symptoms and nonmotor symptoms, including cognitive decline, depression, visual hallucinations, rapid eye movement sleep behavior disorder, excessive daytime sleepiness, and gait freezing.^22^, ^23^, ^24^, ^25^, ^26^, ^27^ Similarly to our previous study that increased FC in DMN regions (right PreCUN and right ANG) was positively correlated with the severity of PD,^22^ we found increased EC from the CAU.R to the DMN (MTG.R) was positively correlated with motor symptoms, nonmotor symptoms, and depression severity in the PD‐DR group, which indicated that abnormal DMN is involved in the development of drooling in PD. The lingual gyrus (LING) is a part of the occipital cortex that processes visual information and facilitates spatial orientation. In addition to abnormal internal function of the occipital lobe, PD patients with hallucinations had aberrant FC between the LING and other brain regions.^28^, ^29^ Thus, LING may be implicated in the mechanism of visual hallucinations in PD. Various clinical investigations suggested that drooling in PD may be strongly connected with hallucinations.^30^, ^31^ Our findings showing reduced FC from the CAU.R to the LING.L in droolers provide further support to the notion that droolers are more likely to experience hallucinations. Taken together, these results suggest that these reciprocal EC between the CAU.R and cortical regions likely contribute to the disease severity, emotion, cognition, and hallucinations of droolers, which further support that abnormal connectivity in the cortico‐striatal network leads to drooling in PD.

The anterior cingulate and paracingulate gyri (ACG) are mainly involved in cognitive and affective regulation, which has been implicated in cognitive impairment and depression.^32^, ^33^, ^34^ Wang et al.^33^ found increased FC between the right hippocampal formation and the ACG.L in PD patients with mild cognitive impairment compared to those without mild cognitive impairment. However, Feng et al.^34^ discovered reduced FC of the ACG in Alzheimer's disease. As the disease progressed, all internet connections might decrease. Here, the decreased FC from the CAU.R to the ACG.L in droolers might help facilitate further understanding of the potential mechanisms underlying cognitive impairment in droolers. Moreover, we discovered that droolers had a decreased EC from the CAU.R to the CB.R relative to healthy controls. The cerebellum is a significant subcortical structure that influences multiple aspects of motor, cognitive, and affective behavior.^35^ Reduced FC between the putamen and cerebellum was linked with the severity of PD.^36^ Such a result may indicate that weakened striatal‐cerebellar connections are a manifestation of disrupted brain network interactions in droolers. Interestingly, increased EC from the CB.L to the CAU.R was observed in droolers relative to non‐droolers. Previous studies have found that cerebellum activation was discovered during autonomic swallowing and in PD patients with dysphagia.^11^, ^37^, ^38^ While it is difficult to establish conclusive interpretations of our results, the FC abnormalities between the CAU.R and cerebellum may be a sign of impaired swallowing and cognitive function in droolers.

### 4.2 EC from and to PoCG.R

Saliva swallowing involves high activation likelihood in the sensorimotor cortex and bilateral cingulate gyrus.^39^ The precentral gyrus is responsible for initiating oropharyngeal and tongue movements, while the postcentral gyrus is engaged in processing oropharyngeal sensory information during swallowing. Functional imaging studies related to normal saliva/water swallowing activity and swallowing disorders further confirm abnormalities in the PoCG neural network.^39^ The fMRI results confirmed that PD patients with dysphagia showed greater activation in the precentral gyrus and supplementary motor area.^37^ Therefore, the enhanced connections from the PoCG.R to the PreCG.L in the PD‐DR group may play a crucial role in salivary swallowing. These similarly affected brain regions may reveal that dysphagia mainly contributes to the experience of drooling in PD. Similarly, the putamen performs a crucial role in sensorimotor coordination. Hou et al.^6^ reported that the posterior putamen had significantly reduced FC with bilateral sensorimotor areas in droolers compared with non‐droolers and healthy controls. We also observe a significantly increased causal connectivity from the PoCG.R to the PUT.L in droolers. This may reflect the natural progression of PD. The posterior putamen, which experiences more extensive damage in early PD, may exhibit a decrease in FC, while unaffected areas of the striatum (anterior putamen and caudate nucleus) may present an increase in FC.^8^

The MTG has been linked to a wide range of functions such as language processing, observation of motion, dynamic facial expressions, and deductive reasoning.^40^ As mentioned previously, MTG is a component of the DMN, as is ORBsupmed.R located in the frontal cortex. The decreased EC from PoCG.R to MTG.R and increased EC from ORBsupmed.R to PoCG.R provide additional evidence that DMN function is compromised in droolers, which may contribute to the “drooling network” in PD. Additionally, we found that EC from the ROL.R to the PoCG.R was reduced in droolers compared with non‐droolers. Rolandic operculum (ROL) is involved in speech production and motor control.^41^ PD patients have been reported to have structural or functional changes in the Rolandic operculum (ROL).^42^, ^43^ In early PD, reduced ReHo values in the ROL are most likely connected to swallowing and speech disorders.^44^ In patients with amyotrophic lateral sclerosis, gray matter atrophy in the ROL is associated with movement disorders or tonic contractions of the perioral muscles, which could induce swallowing dysfunction or dysarthria.^45^ These results demonstrate that droolers may experience dysarthria or swallowing difficulty due to alterations in ROL function.

Several limitations of the present study should be acknowledged. First, this study was a cross‐sectional study and no follow‐up study was conducted to analyze the dynamic changes in brain functions in droolers and non‐droolers. Second, the sample size of the study was not large, and there may be statistical bias. Third, although all subjects stopped taking anti‐PD drugs for at least 12 h before undergoing an imaging scan, it is not fully determined whether long‐term drug use could potentially affect the experimental results.

In conclusion, this study discovered that PD patients with drooling have changes in the cortico‐limbic‐striatal‐cerebellar and cortio‐cortical networks, which might reflect that droolers have abnormal brain integration functions, neurological, and downregulation or compensation mechanisms in specific areas. Drooling disrupts the information flows in the cortico‐striatal, cerebellar‐striatal, limbic‐striatal, and cortio‐cortical loops of PD patients. These FC abnormalities in extensive brain regions not only form a “drooling network” in PD, but may also be used to explain the neural underpinnings of cognitive impairment, hallucinations, depression, dysphagia, dysarthria, and other symptoms in PD patients with drooling. Moreover, abnormal FC could predict the severity of motor symptoms, nonmotor symptoms, and depression in droolers and improve the discrimination between non‐droolers and non‐droolers. This study provides new ideas for further exploring the neuropathological mechanism of drooling in PD.

## AUTHOR CONTRIBUTIONS

YDZ, YYT, CYC, and TH were involved in study design and manuscript draft. TH, LLT, and YJZ were involved in recruitment of subjects and data collection. TH, LLT, YJZ, and SAS carried out data analysis and discussion. All authors have read and approved the final manuscript.

## FUNDING INFORMATION

This work was supported by the Natural Science Foundation of Jiangsu Province (BK20201117), National Science and Technology Innovation 2030—Major program of “Brain Science and Brain‐Inspired Intelligence Research” (2021ZD0201807), and Medical Research Project of Jiangsu Provincial Health Commission (M2022012).

## CONFLICT OF INTEREST STATEMENT

The authors have declared that no conflict of interest, financial or otherwise, exists.

## Supporting information


Supplementary Materials

## Data Availability

All data generated or analyzed during this study are included in this published article and its supplementary information files.
