# Supplementary material for: Drooling disrupts the brain functional connectivity network in Parkinson's disease
Source: CNS Neurosci Ther. 2023 May 5;29(10):3094–107. doi: 10.1111/cns.14251 (PMC10493659; doi:10.1111/cns.14251)
Supplement: Supplementary file 1 — Supplementary Materials [file CNS-29-3094-s001.docx]

**SUPPLEMENTARY MATERIALS**

**ALTERED BRAIN FUNCTIONAL CONNECTIVITY NETWORK IN PARKINSON'S DISEASE WITH DROOLING**

**SUPPLEMENTAL METHODS**

## *1. Exclusion criteria for all the participants*

The exclusion criteria for all the participants were as follows: (1) atypical or secondary parkinsonism; (2) other brain injuries, neurologic or psychiatric illness (traumatic brain injury, ischemic/hemorrhagic stroke, Alzheimer's disease, epilepsy, and schizophrenia); (3) visual hallucination; (4) cognitive impairment: MMSE ≤ 24 for patients with education higher than junior middle school; MMSE ≤ 20 for primary school; MMSE ≤ 17 for illiteracy; (5) moderate to severe head tremor; (6) any condition contraindicating MRI scanning (e.g. incompatible metal implants, pacemakers, arterial stents, and claustrophobia).

## *2. Details of this study design*

For Parkinson's disease with drooling (PD-DR) and Parkinson's disease without drooling (PD-NDR) groups, all patients were subjected to identical experimental steps in the "off" medication state (≥ 12h after the last dopaminergic medication). The demographic and clinical data, including age, gender, handedness, education, disease duration, and levodopa equivalent daily dose (LEDD) were collected after MRI scanning. Meanwhile, PD patients were evaluated by clinical assessments during the medication off phase, including modified Hoehn-Yahr stage (H-Y stage), Movement Disorder Society-Unified Parkinson's Disease Rating Scale (MDS-UPDRS), Non-Motor Symptom Scale (NMSS), Sialorrhea Clinical Scale for Parkinson's disease (SCS-PD), Mini-Mental State Examination (MMSE), Montreal Cognitive Assessment (MOCA), Hamilton Depression Scale (HAMD), and Hamilton Anxiety Scale (HAMA). For the HCs group, each individual was administered the cognitive assessments (MMSE and MOCA). MDS-UPDRS and H-Y stage were applied to evaluate the severity of functional disability associated with PD. The severity of nonmotor symptoms was assessed by the NMSS, whereas the severity of drooling was evaluated using item number 19 of NMSS and SCS-PD. MMSE and MOCA were administered to estimate cognitive performance. In addition, HAMD and HAMA were used to assess the severity of depression and anxiety, respectively.

## *3. Sequence parameters*

Functional imaging data based on BOLD were acquired using a gradient echo-planar imaging (EPI) sequence with the following parameters: repetition time (TR) = 2000 ms, echo time (TE) = 30 ms, slices = 33, thickness = 4 mm, gap = 0 mm, field of view (FOV) = 192 mm × 192 mm, acquisition matrix = 64 × 64, and flip angle (FA) = 90°. Simultaneous multi-slice (SMS) reconstruction was used. All subjects wore earplugs (Hearos Ultimate Softness Series, USA) to protect against the scanner noise, and their heads were immobilized with foam padding to minimize movement artifacts. During the scanning, subjects were instructed to stay awake with their eyes closed, without thinking about anything specific, and to avoid falling asleep.

## *4. Data preprocessing procedure*

The first 10 time points of fMRI data were discarded to account for signal equilibrium and participant adaptation. Then, the slice-timing correction and realignment were performed for the remaining 220 consecutive images. Five PD patients and two healthy individuals were excluded due to severe head motion during MRI scanning (> 2.0 mm translation or 2.0°rotation). After that, the remaining data were spatially normalized to the Montreal Neurological Institute (MNI) template and resampled to 3 × 3 × 3 mm voxels. Finally, linear detrend and temporal filtering (0.01-0.08 Hz) was performed to reduce the effects of low-frequency drifts and high-frequency physiological noise. In addition, the spurious covariates related to the effects of head motion, global mean, white matter (WM), and cerebrospinal fluid (CSF) signals were also removed by a linear regression process.

## *5. The specific steps of independent component analysis (ICA)*

Group ICA decomposition was performed with 27 components using the Infomax algorithm following a repeated 100 times analysis with ICASSO ^1^. ICA consists of three major steps: (1) data reduction, (2) group ICA, and (3) back reconstruction. The intensity values in each independent component were transformed to Z scores to display the voxels which are most strongly relevant to a particular IC. Subsequently, two-sample t-tests were conducted at the group level to investigate the different brain regions between the PD-DR and PD-NDR groups within the SMN mask generated from the results of one-sample t-tests. A threshold of corrected cluster *p* < 0.05 (single voxel *p* < 0.01, cluster size > 40 voxels) was set.

## *6. GCA analysis*

The time series for the CAU.R and the PoCG.R were respectively designated as the seed time series x, while the time series of all voxels in the brain were defined as y. The signed path coefficients were subsequently calculated as GCA values, including the linear direct effect of x on y (Fx→y) and y on x (Fy→x). Therefore, two Granger causality maps were constructed based on each subject's influence measures. Finally, the GCA maps were converted to z-values maps using Fisher’s r-to-z transformation to improve normality.

**REFERENCES**

1. Himberg J, Hyvärinen A, Esposito F. Validating the independent components of neuroimaging time series via clustering and visualization. *NeuroImage.* 2004;22(3):1214-1222.

**SUPPLEMENTAL TABLES**

**Table S1. Correlations between abnormal EC and clinical characteristics of PD patients with drooling and PD patients without drooling before and after correction for age, sex, and education.**

|  | Before correction | After correction |
| --- | --- | --- |
| Correlations between abnormal effective connectivity and PD-DR characteristics | | |
| MDS-UPDRS score and MTG.R | r = 0.562, p = 0.008* | r = 0.598, p = 0.009* |
| MDS-UPDRS II score and MTG.R | r = 0.514, p = 0.017* | r = 0.535, p = 0.022* |
| NMSS score and MTG.R | r = 0.529, p = 0.014* | r = 0.504, p = 0.033* |
| HAMD score and MTG.R | r = 0.513, p = 0.017* | r = 0.523, p = 0.026* |
| MDS-UPDRS score and IPL.R | r = 0.473, p = 0.03* | r = 0.642, p = 0.004* |
| Correlations between abnormal effective connectivity and PD-NDR characteristics | | |
| MOCA score and PCUN.L | r = 0.446, p = 0.03* | r = 0.616, p = 0.005* |

*Significant difference, *p* < 0.05. Partial correlations were corrected for age, sex, and education. PD, Parkinson's disease; PD-DR, Parkinson's disease with drooling; PD-NDR, Parkinson's disease without drooling; MDS-UPDRS, Movement Disorder Society-Unified Parkinson's Disease Rating Scale; MTG.R, right middle temporal gyrus; MDS-UPDRS II, Movement Disorder Society-Unified Parkinson's Disease Rating Scale Part II score; NMSS, Non-Motor Symptom Scale; HAMD, Hamilton Depression Scale; IPL.R, right inferior parietal lobe; MOCA, Montreal Cognitive Assessment; PCUN.L, left precuneus gyrus.

**Table S2. The AUC, 95% CI, P. value, optimal cutoff value, sensitivity, specificity, and Youden index of EC values for distinguishing PD patients with drooling from those without drooling.**

| **Brain region (AAL)** | | **AUC** | **95% CI** | ***p*. value** | **Optimal cutoff value** | **Sensitivity** | **Specificity** | **Youden index** |
| --- | --- | --- | --- | --- | --- | --- | --- | --- |
| **EC from right caudate nucleus** | | | | | | | | |
| Right middle temporal gyrus | 0.864 | | 0.750-0.978 | 0.000* | 0.479 | 0.714 | 0.955 | 0.669 |
| Right inferior parietal lobe | 0.805 | | 0.677-0.933 | 0.001* | 1.573 | 0.571 | 0.909 | 0.481 |
| Left anterior cingulate and paracingulate gyri | 0.905 | | 0.813-0.997 | 0.000* | 0.32 | 0.909 | 0.810 | 0.719 |
| **EC to right caudate nucleus** | | | | | | | | |
| Left cerebellum | 0.909 | | 0.826-0.992 | 0.000* | -0.323 | 0.857 | 0.818 | 0.675 |
| Right inferior parietal lobe | 0.816 | | 0.681-0.951 | 0.000* | 0.09 | 0.773 | 0.857 | 0.630 |
| **EC from right postcentral gyrus** | | | | | | | | |
| Right middle temporal gyrus | 0.836 | | 0.718-0.953 | 0.000* | 0.33 | 0.682 | 0.857 | 0.539 |
| **EC to right postcentral gyrus** | | | | | | | | |
| Right Rolandic operculum | 0.823 | | 0.697-0.948 | 0.000* | 0.36 | 0.727 | 0.905 | 0.632 |

*Significant difference, *p* < 0.05. AUC, area under the receiver operating characteristic curve; CI, Confidence interval; EC, effective connectivity; PD, Parkinson's disease.

**SUPPLEMENTAL FIGURE**


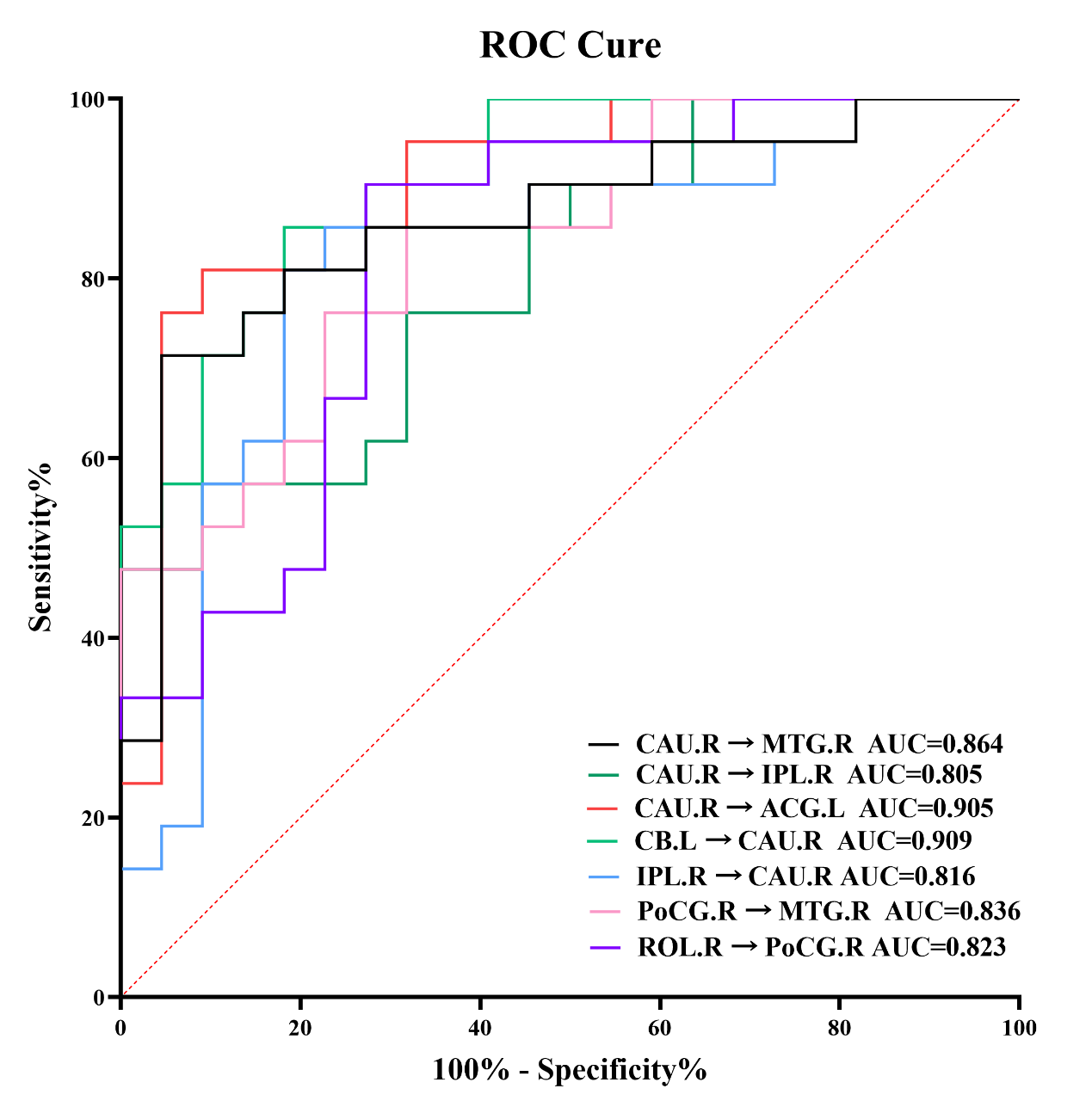


**Figure S1. ROC analysis of EC values for distinguishing PD patients with drooling from those without drooling.** CAU.R, right caudate nucleus; MTG.R, right middle temporal gyrus; IPL.R, right inferior parietal lobe; ACG.L, left anterior cingulate and paracingulate gyri; CB.L, left cerebellum; PoCG.R, right postcentral gyrus; ROL.R, right Rolandic operculum; AUC, area under the ROC curve; ROC, receiver operating characteristic; EC, effective connectivity; PD, Parkinson's disease.
